# The “Crumble Disc Sign”: A Specific MRI Sign of Intradural Lumbar Disc Herniation, Allowing a Preoperative Diagnosis

**DOI:** 10.5334/jbr-btr.910

**Published:** 2015-12-30

**Authors:** Patrick Mailleux, V. Marneffe, Ives Michel, Jean-Philippe Dehullu

**Affiliations:** 1 Clinique St Luc Bouge, BE; 2 Clinique d’Ottignies, BE

**Keywords:** intradural disc herniation, spinal surgery, epidural injection

## Abstract

Intradural disc herniation (IDH) is very rare. Most diagnoses were surgical but with the improvements in magnetic resonance imaging (MRI) it has become possible to preoperatively diagnose intradural migration of disc in some cases. We report a case with very specific imaging features in MRI, well correlated with the surgical findings.

## Introduction

Intradural disc herniation (IDH) is a very rare complication of intervertebral disc hernia, accounting for 0.3–1.5% of all disc herniations [1]. It is frequently associated with symptoms worse than the regular disc herniation [2,3]. Since the first description by Dandy in 1942, over a hundred cases have been described in the literature. We report a case where the MRI images were very specific, allowing a preoperative diagnosis. The MRI examen could demonstrate the precise entry point of the herniation through the dura mater, the large disc fragment descending into the thecal sac, with a typical “crumble” appearance, probably related to the fragmenting effect of the cerebrospinal fluid on the nucleus pulposus substance.

## Case Report

The patient is a 66 years old male patient who had prior spine surgery 15 years ago for a right L4-L5 disc herniation with initial good clinical results. Three years later, he complained of pain recurrence in the right L5 territory. Imaging at that time showed no new disc herniation and he was referred to the pain clinic. Several intra-foraminal L4-L5 and L5-S1 corticosteroid injections were performed during the last 10 years, as well as percutaneous radiofrequency denervation at the L4-L5 and L5-S1 facet joints. Long term complains were moderate.

In June 2014, the patient complained of a very severe bilateral L5 sciatalgy with paresthesias but no motor dysfunction. Mictional delay was also reported.

**Figure 1:**
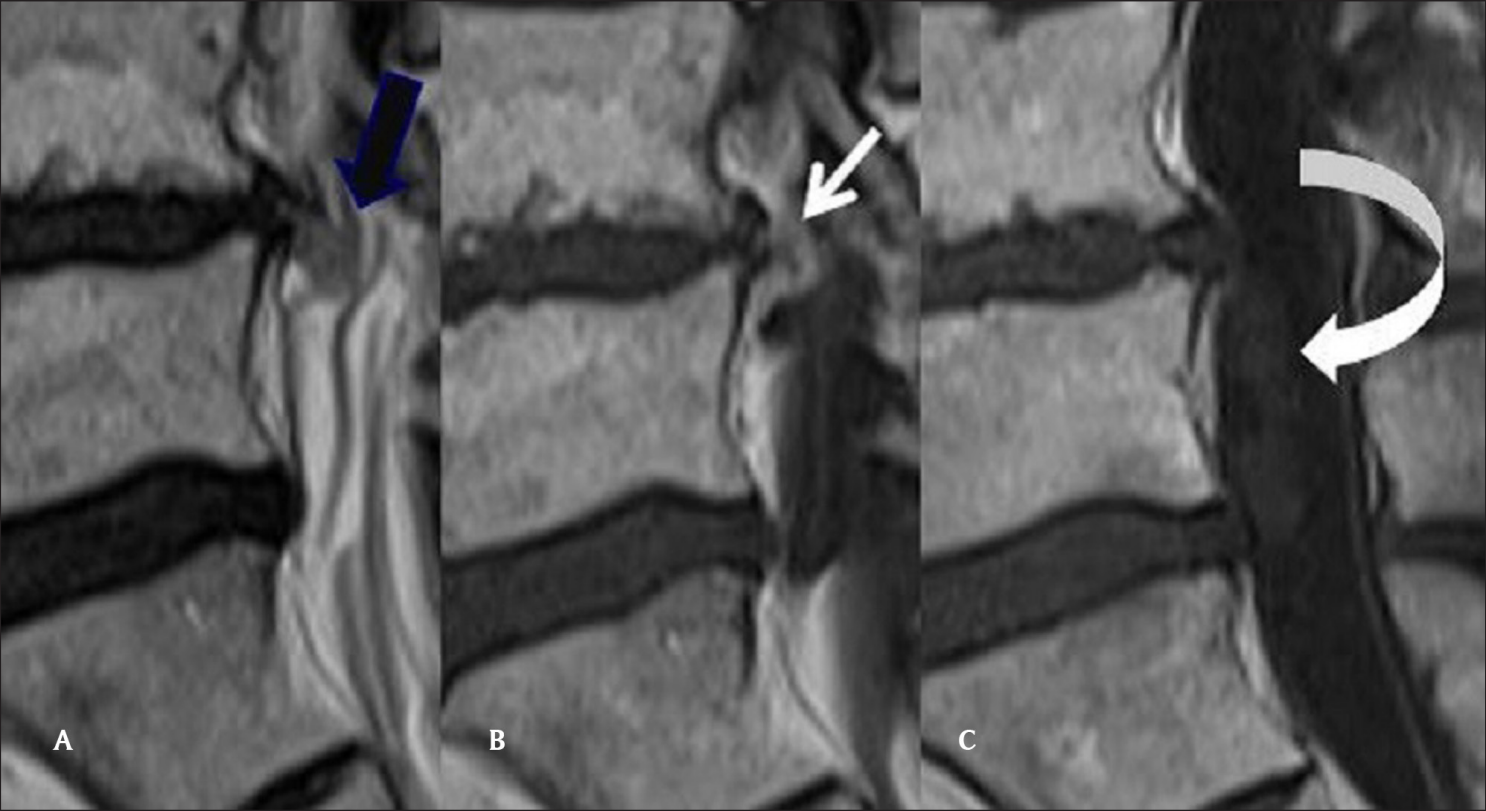
**(A)** Sagittal T2 Mri of the lumbar spine: Continuity of the disc and the upper part of the disc herniation (back arrow). **(B)** Sagittal T1 Mri of the lumbar spine, after gadolinium injection. The upper part of the disc is compact with peripheral contrast enhancement (white arrow). **(C)** Sagittal T1 Mri of the lumbar spine, after gadolinium injection. The intradural component of the IDH does not enhance (curved arrow).

**Figure 2A,B:**
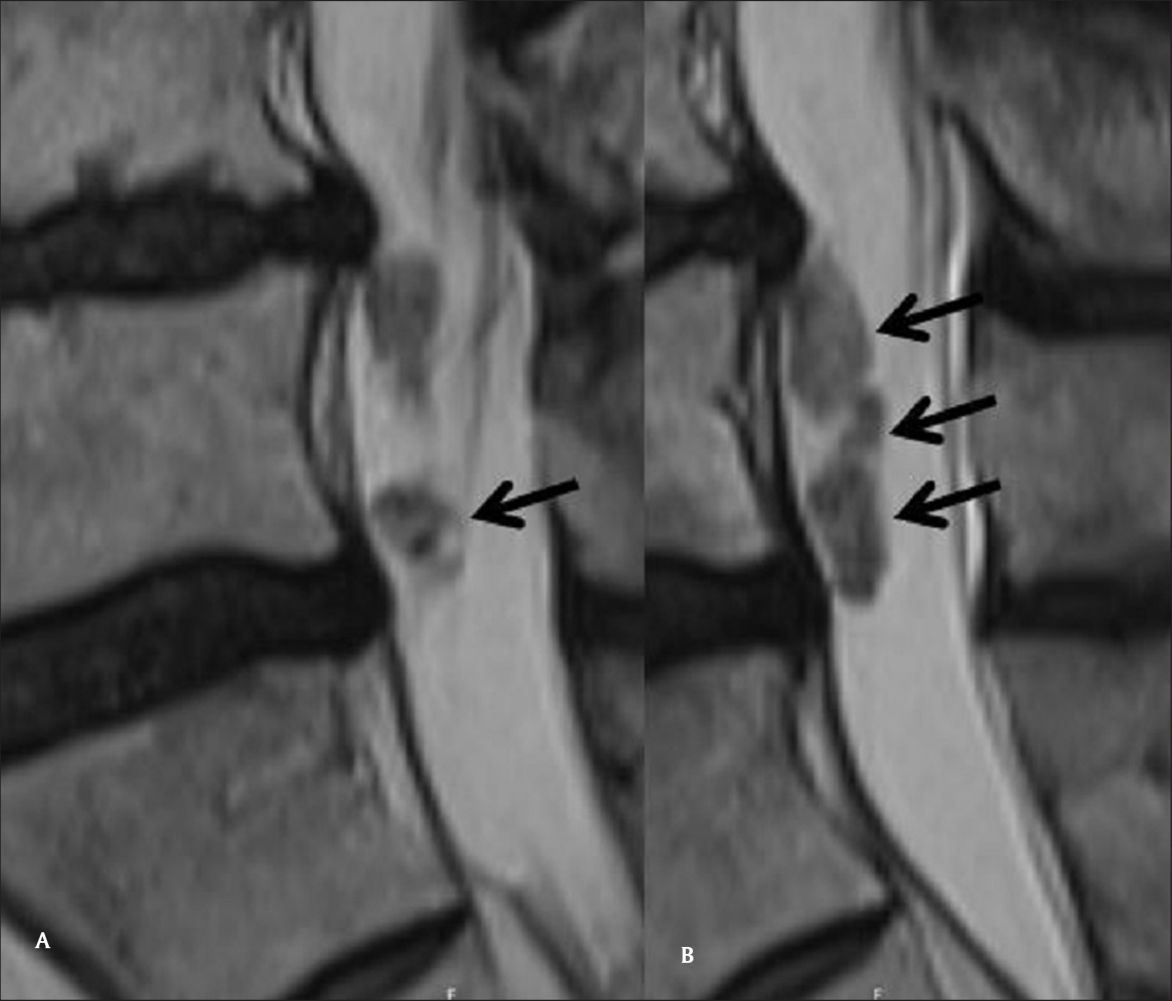
Sagittal T2 Mri of the lumbar spine, “crumbled” aspect of the intradural component of the IDH (arrows).

**Figure 3A–D:**
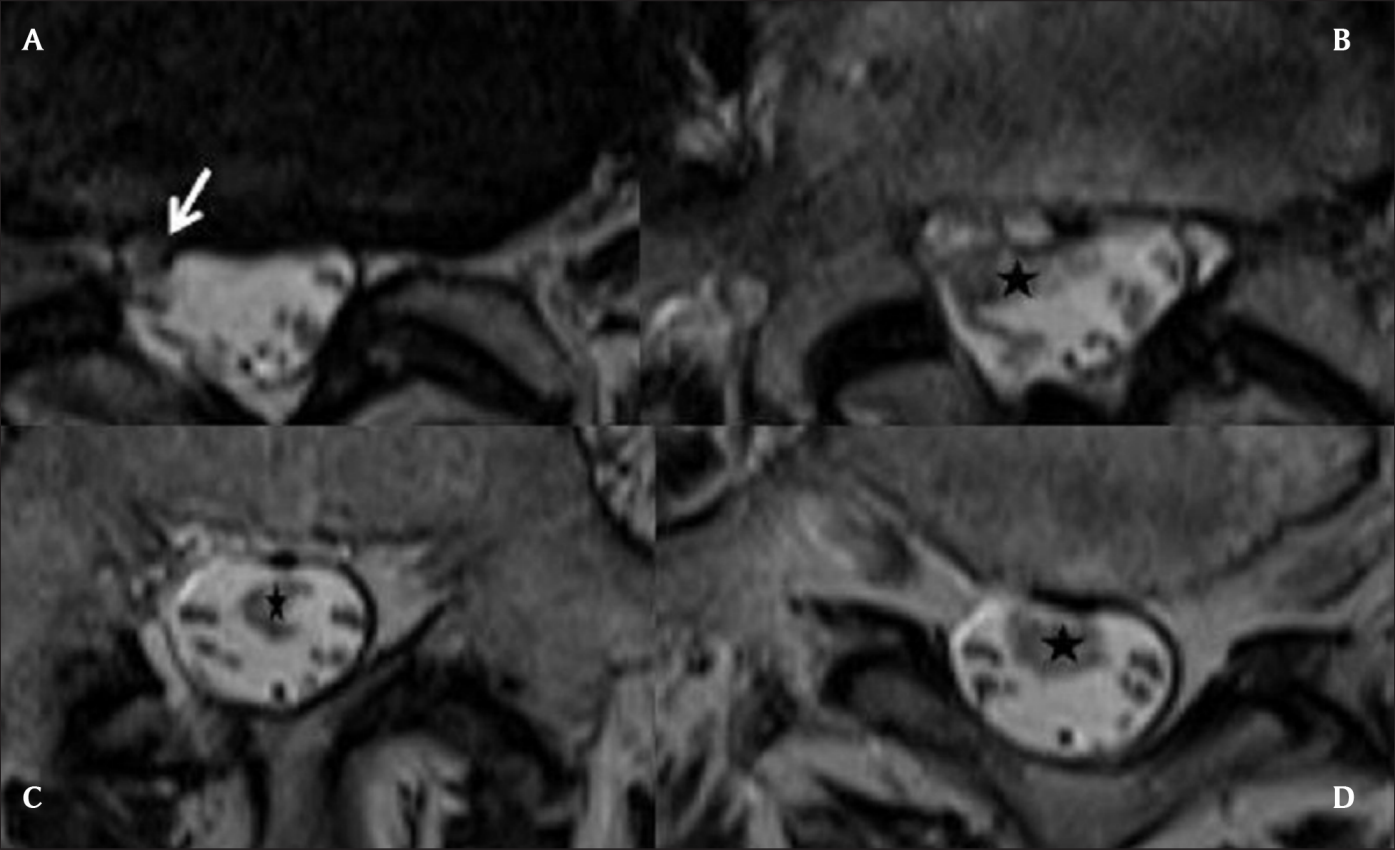
Axial T2 slices of the lumbar spine, from the level of the L4-L5 disk to the sacrum. The IDH enters at the L4-L5 level (white arrow) and extends downwards in the CSF, to the L5-S1 level (black stars). It has irregular borders, heterogeneous nodular structure and variable diameter.

MRI performed in July 2014 shows a disc herniation originating from the L4-L5 disc. It perforates the common longitudinal ligament and the anterior aspect of the dura, and enters the thecal sac (Fig. 1A and 1B), The intradural “part” of the herniation is very large and extends downwards (Fig. 2A and B), reaching the level of the upper sacrum, the disc fragment being surrounded by the rootlets (Fig. 3A to Fig. 3D). The upper part of the disc fragment, at the level when it enters the dura, is “compact”, as usually seen in extradural disc herniations (Fig. 1A), and some peripheric contrast enhancement can be seen after gadolinium injection, while the intradural part seems to be less compact, with a “crumbled” appearance (Fig. 2A and 2B), irregular borders and no contrast enhancement.

Surgery was performed in prone, antilordotic position under general anesthesia, via hemilaminectomy L4 and L5 on the clinically dominant right side with adjacent hemiflavectomy L3/4 and L5/S1, making a sharp parasagittal dural opening possible from a normal aspect of the dural sac into a heavily, scar tissue related metaplastic part of it (as often seen in reoperations and accountable for a higher risk of dural leak). The herniation was plain to see inside the dural sac, clearly causing a compression of most adjacent rootlets, but not really adherent to them (maybe due to the contact of cerebro-spinal fluid).

**Figure 4A,B:**
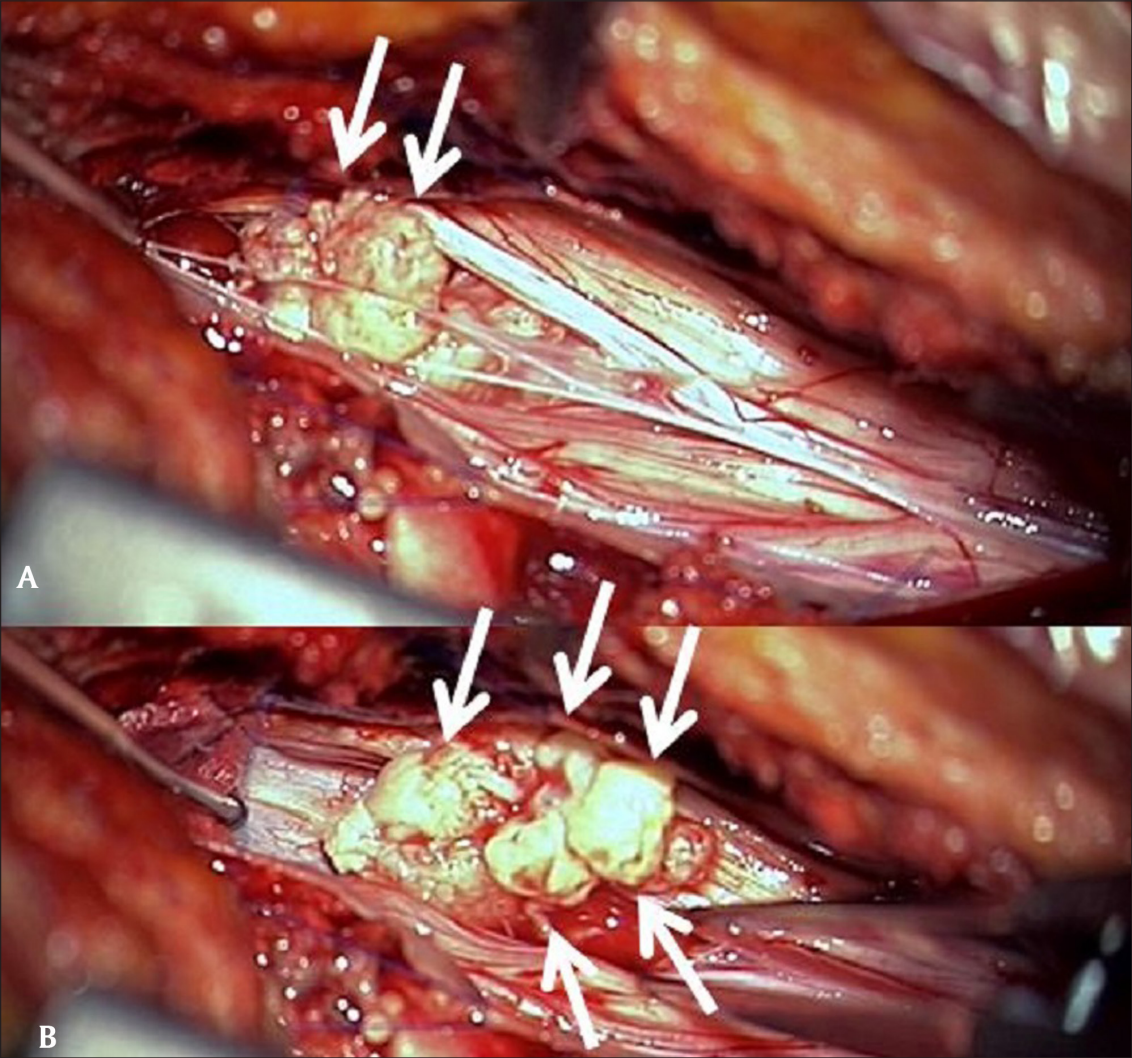
Peroperative views after parasagittal opening of the dura. Multiple nodular fragments of a spongious disc herniation are extracted.

The neurosurgeon found the disc fragment in the spinal fluid to be “spongious, more friable and softer than the usual extradural herniation (Fig. 4A and 4B).

Patient was discharged asymptomatic on day 5 and had an uneventful recovery.

## Discussion

Although an exact pathogenesis for lumbar IDH has not been yet determined, it is generally accepted that adhesions between the annulus fibrosus, posterior longitudinal ligament (PLL), and ventral aspect of the dura represent a predisposing factor. IDH is more frequent if the patient had previous lumbar spine surgery. In our case, the patient had lumbar surgery 15 years earlier, and surgery was followed by many percutaneous procedures in this area (periradicular corticosteroid injections, facet joint denervation). Those could have had a scarring effect while the cortisteroid injections could have fragilized the posterior longitudinal ligament and the dura.

IDH can be divided into intraarachnoid (as in our patient) and extraarachnoid. In the case of the less frequent intradural extraarachnoid disc herniation, the arachnoid is peeled from the dura by the disc herniation and the disc tissue enters this virtual space, but does not reach the rootlets and the CSF [4].

Preoperative diagnostic of IDH was rare, but MRI with gadolinium injection can be helpful when it reveals the ‘hawk-beak’ sign [1], which is composed of a beak-like mass that experiences ring enhancement at the intervertebral space. An abrupt loss of continuity of the posterior-longitudinal ligament on MRI is also suggestive of lumbar IDH, and thought to indicate the portion of the ligament that was transversed by the disc fragment.

Some IDH demonstrate peripheral disc enhancement on Gd-MRI [3]. The rim enhancement was attributed to granulation tissue around the avascular disc fragment, which was confirmed on histopathology. Since then many authors have demonstrated ring enhancement of intradural disc fragments, differentiating it from a variety of intradural tumors [3] that present with progressive leg pain, back pain or neurological deficits and can often be differentiated on clinical symptoms.

While in acute disc herniation, contrast enhancement will usually not be seen [2], the granulation tissue that forms around the disc fragment being a chronic process.

Our patient’s disc herniation presented several specific features, allowing diagnosis : there is no discontinuity between the intervertebral disc and the disc fragment penetrating the dura (Fig. 1A), there is some peripheral enhancement at this location (Fig. 1B), but the intradural part does not enhance ( as would meningiomas and neurinomas : Fig. 1C), and has a « crumbled « appearance, a little bit enlarged compared to the other part, with irregular borders and a spongious inner structure (Fig. 2A and 2B). This structure floats between the roots, inside the dural sac (Fig. 2B and Fig. 3).

This spongious heterogeneous appearance is thought to be caused by the action of the cerebrospinal fluid on the disc, having a fragmenting effect on disk tissue not compressed anymore by the adjacent structures. And that was the clinical finding of the surgeon. We think that this « crumble disc sign », while quite unfrequent, is a very specific MRI sign for the intradural localization of the disc herniation.

## Competing Interests

The authors declare that they have no competing interests.
